# Nanoscale simultaneous chemical and mechanical imaging via peak force infrared microscopy

**DOI:** 10.1126/sciadv.1700255

**Published:** 2017-06-23

**Authors:** Le Wang, Haomin Wang, Martin Wagner, Yong Yan, Devon S. Jakob, Xiaoji G. Xu

**Affiliations:** 1 Department of Chemistry, Lehigh University, 6 East Packer Avenue, Bethlehem, PA 18015, USA.; 2 Bruker Nano, 112 Robin Hill Road, Santa Barbara, CA 93117, USA.; 3 Department of Chemistry and Environmental Science, New Jersey Institute of Technology, Newark, NJ 07102, USA.

**Keywords:** Super-resolution imaging, infrared microscopy, chemical imaging, nanoscale characterization, mechanical mapping, Block copolymer, scanning probe microscopy, peakforce tapping

## Abstract

Nondestructive chemical and mechanical measurements of materials with ~10-nm spatial resolution together with topography provide rich information on the compositions and organizations of heterogeneous materials and nanoscale objects. However, multimodal nanoscale correlations are difficult to achieve because of the limitation on spatial resolution of optical microscopy and constraints from instrumental complexities. We report a novel noninvasive spectroscopic scanning probe microscopy method—peak force infrared (PFIR) microscopy—that allows chemical imaging, collection of broadband infrared spectra, and mechanical mapping at a spatial resolution of 10 nm. In our technique, chemical absorption information is directly encoded in the withdraw curve of the peak force tapping cycle after illumination with synchronized infrared laser pulses in a simple apparatus. Nanoscale phase separation in block copolymers and inhomogeneity in CH_3_NH_3_PbBr_3_ perovskite crystals are studied with correlative infrared/mechanical nanoimaging. Furthermore, we show that the PFIR method is sensitive to the presence of surface phonon polaritons in boron nitride nanotubes. PFIR microscopy will provide a powerful analytical tool for explorations at the nanoscale across wide disciplines.

## Teaser

A new scanning probe microscopy images the nanoscale world with synergistic force and infrared light at 10-nm spatial resolution.

## INTRODUCTION

Properties of materials depend on the composition and organization of their constituent components at the small scale. In a variety of disciplines of nanoscience and nanotechnology, characterizations of the chemical compositions, mechanical properties, and geometric shapes with tens of nanometers of spatial resolution are an essential part of everyday research. A nondestructive, ambient-compatible, spectromicroscopy method that can deliver chemical-sensitive imaging, the broadband spectrum from the nanoscale, together with the topographic and mechanical properties with a spatial resolution of ~10 nm, is highly desirable for the broad research communities. These joint characterization abilities would undoubtedly accelerate the research across a broad range of research fields. However, current analytical techniques are insufficient to provide the desired multimodal characterization abilities.

The material response to infrared light is indicative of its chemical composition because infrared absorptions directly correspond to the vibrational modes of functional groups in chemical materials and lattice vibrations in crystals. However, the spatial resolution of conventional Fourier transform infrared (FTIR) microscopy is bound by the optical diffraction limit to half of the wavelength (*1*), which corresponds to several micrometers. Consequently, nanoscale features are not resolvable spatially in conventional far-field infrared microscopy while the spectra suffer from inhomogeneous line broadening due to ensemble averaging within the large sampling area. Popular fluorescence-based super-resolution techniques (*2*–*4*) are not applicable to the mid-infrared range due to the lack of suitable labels. The limited resolution improvement factor (that is, approximately a factor of 2) from the structural illumination technique (*5*) is insufficient for infrared microscopy to reach the nanoscale.

The combination of scanning probe microscopy with infrared illumination provides ways to beat the diffraction limit at mid-infrared frequencies. One of these techniques, infrared scattering-type scanning near-field optical microscopy (s-SNOM), uses a sharp metal-coated probe position-controlled by an atomic force microscope (AFM) to locally enhance the optical field and to excite the vibrational (*6*) phonon (*7*) or polaritonic resonances in the sample (*8*, *9*). Subsequently, the high–spatial frequency optical near field generated or attenuated by the presence of the sample is probed locally by the sharp probe, thus providing spatial contrast better than the diffraction limit of the propagating optical field. The typical spatial resolution of s-SNOM is determined by the radius of curvature of the probe, which is usually 10 to 20 nm. However, s-SNOM has limitations as an analytical technique. First, the elastic light scattering process of s-SNOM means that a considerably large far-field background is present because of incident light scattered from the surroundings of the sharp apex of the probe. As a result, interferometric detection and lock-in signal demodulations (*10*) are required to obtain the s-SNOM contrast, which adds complexity to the apparatus. Second, the spectral profile from s-SNOM is not based on the absorption of the light field, but rather the scattering efficiency of the incident field by the tip and sample. Therefore, the direct s-SNOM spectra from the near-field amplitude do not correspond to the infrared absorption spectra, which are commonly used for chemical identification. Special double modulation techniques (*11*) or phase-selective homodyne techniques (*12*) are required to obtain the absorption equivalent spectra, which further add complexity. Third, to effectively collect a broad spectrum, broadband infrared sources with Fourier transform–type detection are necessary (*6*). Although there are successful examples of using difference frequency generation from femtosecond lasers (*7*) or synchrotron infrared radiation (*13*, *14*), these light sources are expensive or only limited to certain geographic locations. Compact and less-expensive narrow-band infrared sources such as quantum cascade lasers (QCLs) are not effective for the collection of a broad infrared spectrum with s-SNOM. Last, s-SNOM measures only weak signals from soft matters such as polymers. Hence, only limited examples have been published, mainly on polymers with strong infrared resonances in s-SNOM (*15*).

Action- or force-based infrared microscopy is the other family of infrared microscopy techniques that allow nanoscale infrared imaging and spectroscopy below the diffraction limit. The technique of photothermal induced resonance (PTIR) (*16*–*21*) or photothermal expansion (*22*, *23*) collects the infrared absorption responses through the detection of laser-induced contact resonance or volume expansion in the sample, respectively. They are collectively known as the AFM-IR technique. This type of microscopy method operates with simple instrument designs, as compared to the high complexity of interferometric detection in s-SNOM (*24*). However, the typical spatial resolution of the PTIR techniques operated in contact AFM mode is about 50 to 100 nm. Only for some particular samples, contact mode photothermal microscopy has been demonstrated to provide a spatial resolution of approximately 20 nm (*22*, *25*). A typical spatial resolution of 50 to 100 nm is not enough for detailed nanoscale characterization of samples with small features. The contact mode operation of the PTIR technique also limits its application to smooth and clean samples. Rough or sticky sample surfaces that are not suitable for contact mode AFM operation are also not suitable for PTIR measurements.

A recent innovation of infrared nanoscopy is photoinduced force microscopy (PiFM) through dipole-dipole interactions between the metallic probe and the induced dipoles of the sample (*26*). PiFM operates in tapping mode AFM at one mechanical resonance of the cantilever and detects the motion of the cantilever at the other mechanical resonance with the infrared laser pulsed at a frequency equal to the difference between these two resonances. PiFM allows infrared imaging of polymer surfaces with a spatial resolution of ~10 nm as well as a collection of broadband spectra with a QCL. Although there is a debate on the exact underlying mechanism (*27*), PiFM is a powerful analytical method in comparison to PTIR techniques regarding spatial resolution and the use of tapping mode. The minor limitation of PiFM as an infrared microscopy technique is the requirement of an AFM probe to have two well-behaved mechanical resonances with suitable frequency difference, which may be difficult to find or may require special probe designs.

Nanoscale mechanical properties are an important aspect of material characterization, and AFM has been widely used to study them (*28*, *29*). The nanoscale mechanical properties allow for nanoscale identification of components and provide insight into how the compositions are connected or organized. Although these AFM-based infrared microscopy techniques enable infrared imaging at the nanoscale, none of them are intrinsically suited for simultaneous or correlative measurements of spectroscopic and mechanical information with nanoscale spatial resolution. Thus far, only combinations of sequential utilizations of s-SNOM and peak force tapping–based quantitative nanomechanical mapping (*30*) have been carried out (*31*). Whereas s-SNOM provides spectroscopic information, peak force tapping provides mechanical responses such as modulus and adhesion (*32*). In addition, the precise force control during peak force tapping enables imaging of very delicate samples such as DNA (*33*), and it can be readily combined with potential and conductivity measurements. However, the joint measurement of s-SNOM and peak force tapping requires switching between two operational methods and inherits instrument complexities from both methods. In addition, the scattering-type near-field technique requires the presence of optical near-field enhancement at the apex of the probe, which is vulnerable to contamination or deformation from indentations of the probe in the mechanical measurement. For the same reason, other nanoscale near-field spectroscopic methods, such as tip-enhanced Raman spectroscopy (*34*–*36*) or aperture-based near-field scanning optical microscopy (*37*, *38*), are not suitable for joint mechanical measurement.

Here, we report the development of a novel type of scanning probe microscopy: peak force infrared (PFIR) microscopy. It enables spatial imaging of infrared absorption responses with a spatial resolution of ~10 nm and the collection of broadband infrared spectra from nanoscale locations, together with simultaneous nanoscale mapping of mechanical properties of modulus and adhesion. The method is an action-based spectromicroscopy technique that combines synchronized infrared pulse excitations with the time-gated detection of the laser-induced mechanical responses in the sample. It operates in the peak force tapping mode of AFM (*29*, *30*), which applies to a wide range of samples, including rough, sticky, and very small and delicate samples.

## RESULTS

### Experimental mechanism and signal extraction

**Fig. 1:**
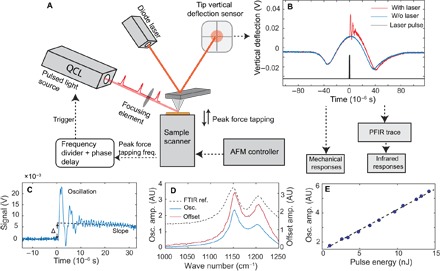
Operational scheme of PFIR microscopy. (**A**) Operation diagram for the PFIR apparatus. Detailed operation mechanisms are described in Materials and Methods. (**B**) Gate-averaged traces of vertical deflections of the cantilever with the laser interaction (red curve) and without the laser interaction (blue curve). The timing of the infrared laser pulse (black curve) is chosen to be within the contact regime of the peak force tapping cycle. Subtraction of the two vertical deflection traces is used to obtain the PFIR trace. (**C**) The PFIR trace of PTFE at the infrared frequency of 1160 cm^−1^ is shown. The laser interaction leads to three types of behaviors: (i) the contact resonance oscillation of the cantilever, (ii) the baseline offset Δ, and (iii) the slope of the baseline. (**D**) PFIR spectra of PTFE from the oscillation amplitude (osc. amp.) (blue curve) and the baseline offset (red curve) across its vibrational resonances. The FTIR spectrum for a bulk sample is included as a reference (black dashed curve). AU, arbitrary units. (**E**) The power dependence of the PFIR oscillation amplitudes for the PTFE sample versus the pulse energy of the infrared laser. A linear dependence is observed.

The schematics of the PFIR apparatus are shown in Fig. 1A, and detailed operational procedures are described in Materials and Methods. In brief, infrared laser pulses from a frequency-tunable QCL are guided to the tip and sample region of an AFM operated in the peak force tapping mode. The peak force tapping mode allows controlled tip-sample contact for each peak force tapping cycle. The laser pulses from the laser are synchronized with every other peak force tapping cycle. The position sensor records time traces of vertical deflections of the cantilever with and without the laser pulse. Figure 1B shows the experimentally acquired cantilever deflection traces in time with infrared laser excitation (red curve) and without laser excitation (blue curve). The absorption of the infrared laser by the sample leads to volume change in the form of thermal expansion. If the volume expansion is rapid enough, then it is capable of excitation of the contact resonance of the cantilever. The expanded volume persists for a certain time before returning to the initial volume due to dissipation of heat through thermal conduction with the surroundings. The difference between the two traces of vertical cantilever deflection is obtained by subtraction, resulting in the PFIR trace. The PFIR trace carries the change of the cantilever motion due to the excitation of the sample by infrared laser. The typical PFIR trace contains three characteristics representing the laser-induced effects: (i) the excitation of the contact resonance (*16*, *39*), (ii) the baseline offset of the PFIR trace that represents the expanded volume due to thermal expansion, and (iii) the change of the slope of the baseline of the PFIR trace after the pulsed laser (it is related to the rate of thermal dissipation through local thermal conductivity or possible changes of modulus after infrared laser absorption). Here, the first and second characteristics are analyzed to represent the magnitude of the infrared absorption to provide the PFIR signal. For brevity, the third PFIR trace characteristic, the slope change, will be the subject of future research. Numerical Fourier transform is used to extract the amplitude of the contact resonance oscillation that is proportional to the local infrared absorption, which is similar to the treatment in PTIR techniques. The baseline offset is calculated by fitting the baseline before and after the laser excitation. Figure 1C shows the PFIR trace taken from the surface of a bulk of polytetrafluoroethylene (PTFE) of 2-mm thickness at the resonant infrared frequency of 1160 cm^−1^. The contact resonance oscillations and the baseline offset (defined as Δ in Fig. 1C) are clearly observed. The baseline offset is found to be 0.3 nm, calculated from the deflection sensitivity of the cantilever. In the spectroscopy operation, the frequency of the infrared laser is scanned, and the amplitude of the contact resonance oscillation or the offset is registered to form the infrared spectra. The amplitude of the contact resonance oscillation is obtained by the Fourier transform of the oscillation region of the PFIR trace and takes the area under the first contact resonance in the frequency domain (fig. S1). Figure 1D shows the infrared spectra obtained from the amplitude of the oscillation (blue curve) and baseline offset (red curve) of the PFIR trace of the PTFE sample. The PFIR spectrum reveals two vibrational resonances of 1160 and 1210 cm^−1^. Far-field FTIR spectrum from the PTFE bulk is displayed (black dashed curve) as a reference, with which the PFIR spectra show excellent agreement. In Fig. 1E, the infrared pulse energy is plotted versus the oscillation amplitude of the PTFE sample at the resonant infrared frequency of 1210 cm^−1^ (a similar plot displaying the pulse energy versus the baseline offset is given in fig. S2). A linear energy dependence is observed, which confirms that the laser-induced mechanical effect is proportional to the incident laser power. Note that the behavior of laser-induced mechanical responses is sample-dependent. The PFIR characteristics of poly(methyl methacrylate) (PMMA; shown in fig. S3) exhibit a more complex energy dependence than those of PTFE, which indicates the presence of a glass transition in the high–laser power regime. The practical implication of the power dependence is that the energy of the pulsed laser source should be kept low within the linear regime to avoid the glass transition when studying polymers.

### Chemical imaging and broadband spectroscopy on polymer and block copolymer

**Fig. 2:**
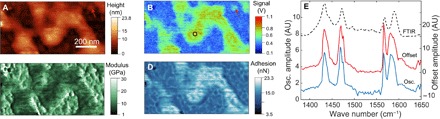
PFIR imaging and spectroscopy on P2VP. (**A**) Topography of nanoscale P2VP polymer islands on a gold substrate. (**B**) The PFIR image of the same area with an infrared frequency of 1433 cm^−1^. Islands of P2VP show increased PFIR response. (**C** and **D**) The measured modulus and adhesion maps of the same area. The mechanical responses are simultaneously collected with the PFIR image. (**E**) PFIR spectra based on the oscillation amplitude of contact resonance (blue curve) and baseline offset (red curve) from the marked locations (black circle) in (B). The FTIR spectrum (dashed curve) is included as a reference.

Figure 2 shows the measurement of poly(2-vinylpyridine) (P2VP) on a gold substrate. The AFM topography image (Fig. 2A) shows that P2VP forms nanoscale dewetted islands. The PFIR image (Fig. 2B) taken at the resonant infrared frequency of 1433 cm^−1^ reveals that the vibrational resonance of P2VP leads to enhanced PFIR signal. In contrast, the substrate lacks infrared resonances, resulting in a small background response. The maps of elastic modulus and adhesion extracted from the Derjaguin-Muller-Toporov (DMT) model (*40*) during peak force tapping are shown in Fig. 2 (C and D). The P2VP polymer shows low modulus and high adhesion compared to the gold substrate, because the polymer is known to be more sticky and softer than the metal surface. Moreover, PFIR microscopy allows the acquisition of the infrared equivalent spectra from a nanoscale location. The PFIR spectra of both the amplitude of contact resonance (blue curve) and the laser-induced baseline offset (red curve) are shown in Fig. 2E. They match the FTIR spectrum (black dashed curve) of the polymer from a separate bulk measurement. The sensitivity of the method can be estimated from the PFIR response from a small polymer island marked by the red arrows in Fig. 2 (A and B). The small island has a thickness of 3 nm and provides obvious signals at a signal-to-noise ratio of 8. We estimate that only 1.1 × 10^3^ (that is, 1.7 zeptomoles) vibrational modes are enough to generate a detectable PFIR signal with a signal-to-noise ratio of 3. This observation suggests that PFIR is a highly sensitive infrared imaging technique. The high signal sensitivity is likely partially due to the presence of enhancement of the infrared field from the gold substrate as well as from the platinum-coated probe. The gap formed between the gold surface and the metallic probe can locally enhance the electromagnetic field to improve the signal (*22*). On the other hand, signals from the thicker area (>20 nm) are stronger than signals from the thin island, which means that the increase of sample thickness improves the signal due to an increased density of vibrational modes, although the field enhancement should be less for the thick area than for the thin area.

**Fig. 3:**
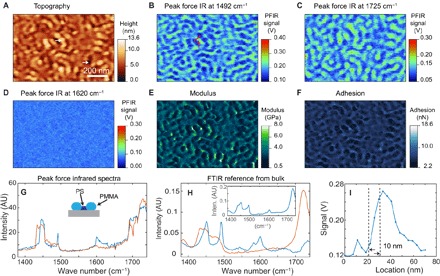
PFIR imaging of nanophase separation of a PS-*b*-PMMA block copolymer. (**A**) Topography of the PS-*b*-PMMA block copolymer. (**B**) PFIR image at the infrared frequency of 1492 cm^−1^, resonant with one of the vibrational modes of polystyrene. Therefore, the domains of polystyrene provide high PFIR signals. (**C**) PFIR at the infrared frequency of 1725 cm^−1^. The infrared frequency is resonantly exciting the vibrational mode of PMMA; therefore, the domains of PMMA provide high PFIR signals. (**D**) PFIR image at the infrared frequency of 1620 cm^−1^. The frequency is off-resonant with both PMMA and polystyrene, so a low PFIR response is observed. (**E** and **F**) Simultaneously registered modulus map (E) and adhesion map (F) of the same region of block copolymer. (**G**) PFIR spectra taken at the two locations marked by the white arrows in (A). The spectrum (red curve) is taken from the ridge of the topography and corresponds to the PMMA domains. The spectrum (blue curve) is taken from the valley of the topography and corresponds to the polystyrene (PS) domain. (**H**) The reference FTIR spectra from the bulk sample of PMMA (red curve), polystyrene (blue curve), and the PS-*b*-PMMA block copolymer (inset). The PFIR spectra show good agreement with the reference FTIR spectra from the bulk. inten., intensity. (**I**) Estimation of the spatial resolution from a section profile in (B) shows a spatial resolution of 10 nm, from the width between 90 and 10% of the edge height of the PFIR signal.

The high spatial resolution of PFIR microscopy allows chemical identification of domains in block copolymers. Block copolymers are a category of structured soft materials that exhibit nanoscale phase separation between the constituted polymer segments (*41*–*43*). Controlled nanoscale phase separation of block copolymers is useful for applications such as lithography templates (*44*). Figure 3A shows the topography of a PS-*b*-PMMA diblock copolymer. Figure 3 (B to D) shows the PFIR images of the block copolymer acquired at the infrared frequencies of 1492, 1725, and 1620 cm^−1^. These PFIR images are registered from the amplitude of the contact resonance oscillation. The PFIR images calculated from the laser-induced baseline offset are shown in fig. S4. The infrared frequency of 1492 cm^−1^ is on resonance with the polystyrene domains, and the infrared frequency of 1725 cm^−1^ is a characteristic of the C=O group present in the PMMA domain. The PFIR images of the two infrared frequencies show complimentary spatial features that correspond to the phase separation of the polystyrene and methyl acrylate domains. The PFIR image taken at the infrared frequency of 1620 cm^−1^ is shown as an example of an off-resonant PFIR image, which does not provide observable signals. The simultaneously collected images of modulus and adhesion of the block copolymer are shown in Fig. 3 (E and F). From the PFIR image at characteristic frequencies, we can identify the domains of the PMMA to be regions of ridges on the topography and those of the polystyrene domain to be shallower trenches in the topography. From the mechanical measurement, the domains of PMMA are associated with comparably low moduli of 2.5 GPa and low adhesions of 3.9 nN. The regions of polystyrene are associated with a slightly higher modulus of about 3 GPa and a higher adhesion of about 5.3 nN. The modulus of the domains is within the literature values of polystyrene and PMMA (*45*). Figure 3G shows the collected PFIR spectra from the two locations on the topography marked by two white arrows in Fig. 3A. The PFIR spectrum from location at the ridge (red curve) corresponds to the spectrum of PMMA, and the PFIR spectrum in the trench (blue curve) corresponds to that of polystyrene. The reference FTIR spectra of PMMA and polystyrene are shown in Fig. 3H, with the FTIR spectrum of the PS-*b*-PMMA block copolymer included in the inset. A profile cut from the PFIR image of Fig. 3B is shown in Fig. 3I, which reveals a spatial resolution of 10 nm.

### Infrared nanoimaging and nanoscale spectroscopy of perovskite nanocrystal

**Fig. 4:**
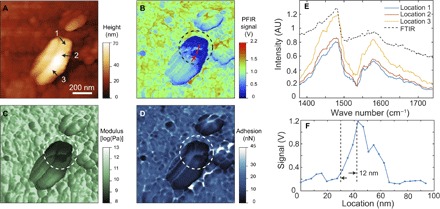
PFIR imaging and spectroscopy on the perovskite crystal. (**A**) The AFM topography of the surface of the CH_3_NH_3_PbBr_3_ perovskite crystal. A 500-nm × 300-nm nanocrystal is observed on the large perovskite crystal surface. (**B**) The PFIR image obtained of the region with the infrared frequency of 1585 cm^−1^. (**C** and **D**) The co-registered modulus and adhesion images of the region on the perovskite crystal. Clear inhomogeneity within the small nanocrystal can be identified from the infrared absorption map, modulus, and adhesion map. (**E**) PFIR spectra from three individual locations [marked as 1, 2, and 3 in (A) on the perovskite nanocrystal]. The FTIR spectrum of bulk perovskite crystals is shown as a reference (black dashed curve). (**F**) A section profile of the PFIR response from the perovskite crystal from the marked red line in (B). It demonstrates a spatial resolution of 12 nm, estimated from the width between 90 and 10% of the height of the signal.

In addition to the straightforward identification of polymers at the nanoscale, PFIR microscopy also allows unambiguous identification of inhomogeneity at the surface of crystals. Perovskite, CH_3_NH_3_PbBr_3_, is an emerging photovoltaic material (*46*). Figure 4A shows a topography image of a perovskite crystal, in which a 500-nm × 300-nm nanocrystal is identified by its large protrusion from the surface. The topographical image does not provide any useful information about this nanocrystal outside of its existence. The PFIR image at the resonant frequency of 1585 cm^−1^ is shown in Fig. 4B. The supporting crystal surface shows rather homogeneous responses. However, the nanocrystal exhibits obvious variation in responses at different locations. The identification of these discontinuities, as noted by large variations in signal strength across the nanocrystal, is a clear advantage of PFIR microscopy over topographical imaging. Simultaneously collected modulus and adhesion images, which further reveal the inhomogeneity of the nanocrystal surface, are shown in Fig. 4 (C and D). The discontinuities of the perovskite nanocrystal are marked by circles in Fig. 4 (B to D).

To study the chemical composition of the nanocrystal at different locations, the PFIR spectra of the three locations (marked as 1 to 3 in Fig. 4A) are collected and shown in Fig. 4E. The two infrared peaks at ~1480 and ~1580 cm^−1^ are from the symmetric and asymmetric bending of the hydrogen atom in the NH_3_^+^ group in the methyl ammonium component of the perovskite crystal (*47*, *48*). During the storage and measurement, CH_3_NH_3_PbBr_3_ was exposed to ambient atmosphere with moderate level of humidity, in which the surface of CH_3_NH_3_PbBr_3_ might have been degraded, which may lead to PbBr_2_. However, the spectral coverage of our laser is not sufficient to directly detect the presence of PbBr_2_. On the other hand, the differences in the intensity of the spectra suggest variations of the density of methyl ammonium at the locations of measurement or by the coverage of other species, as a result of degradation. The degradation of the perovskite crystal and generation of impurities will lead to discontinuity in the crystal associated with the reduction of the mechanical modulus, as observed in Fig. 4C. Increased deformation of the AFM probe during the peak force tapping (fig. S5) also suggests the discontinuity of the crystal structures. The degradation of the perovskite crystal and the presence of discontinuity at the crystal surface may affect the electric property of the material when used as a photovoltaic component. The high spatial resolution of PFIR microscopy allows the in situ detection of the surface inhomogeneity, which would be overlooked by current macroscopic analytical methods. The spatial resolution of the measurement is estimated to be 12 nm from the profile across the region of degradation (Fig. 4F).

### Access to the polaritonic modes of boron nitride nanotubes

**Fig. 5:**
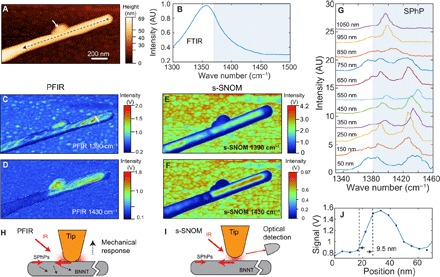
PFIR microscopy studies on BNNT. **A**) The topography of a BNNT on a gold substrate. (**B**) The FTIR spectrum of bulk BNNTs shows the phonon resonances but not the SPhP resonances that would be present above 1380 cm^−1^ marked in the gray box. (**C**) The PFIR image at the infrared frequency of 1390 cm^−1^. (**D**) The PFIR image at the infrared frequency of 1430 cm^−1^. (**E** and **F**) The s-SNOM images taken at 1390 cm^−1^ (E) and 1430 cm^−1^ (F) of the same region of BNNT are shown as references. A similar spatial distribution of the infrared response is observed. (**G**) PFIR spectra taken from a series of locations along the BNNT at 100-nm increments from the tube terminal show position-dependent variations with clear SPhP resonances. The spectra are vertically offset for clarity and were taken along the black dashed line in (A) starting 50 nm away from the terminal. (**H**) A possible SPhP excitation scheme for PFIR. (**I**) The SPhP excitation and detection scheme in s-SNOM. (**J**) Estimation of spatial resolution in the PFIR measurements from a cut profile of the BNNT [white line in (C)] is found to be 9.5 nm from the width between 90 and 10% of the height of the PFIR signal.

The boron nitride nanotube (BNNT) is known to support surface phonon polaritons (SPhPs) in the spectral region of negative dielectric constants above a strong phonon resonance (*49*). In the past, SPhP modes have been studied with s-SNOM that is capable of coupling photons of high momentum to launch SPhPs (*8*, *9*, *49*). We found that PFIR microscopy is also capable of revealing the presence of SPhP in the BNNT. Figure 5A shows the topography of a BNNT. Figure 5B displays the FTIR spectrum from the ensemble measurement of many BNNTs, which marks the absence of the SPhP resonance (>1380 cm^−1^) because it is not accessible by direct far-field infrared radiation. Figure 5 (C and D) shows the PFIR images taken at the infrared frequencies of 1390 and 1430 cm^−1^. Nonuniform distributions of the infrared absorption responses are revealed by the PFIR images. For comparison, s-SNOM images taken at the same frequencies are given for the same nanotube in Fig. 5 (E and F). The distributions of the signals from PFIR and s-SNOM images are quite similar overall; however, the differences in small details are observed. At 1390 cm^−1^, the whole segment of the BNNT shows both s-SNOM and PFIR responses. At 1430 cm^−1^, only a part of the segment shows the s-SNOM and PFIR responses. Detailed examination of the PFIR images reveals the edges of twisting polygonal facets of the BNNT, which were known in the literature (*31*, *50*). In comparison, the s-SNOM measurement in Fig. 5 (E and F) is not capable of resolving them. The defect attached to the BNNT (marked by the white arrow in Fig. 4A) shows a strong PFIR response, although it does not exhibit a strong s-SNOM signal. s-SNOM records a high signal from the gold substrate due to the enhancement of the image dipole of the metallic tip, whereas the substrate response in the PFIR signal is small due to the lack of an absorptive infrared resonance in gold.

The obvious PFIR responses from 1390 to 1420 cm^−1^ above the regular phonon resonance suggest excitation of SPhPs. PFIR spectra taken at 100-nm increments along the BNNT are shown in Fig. 5G from locations marked along the black dashed line in Fig. 5A. The spectral profiles at different locations all exhibit resonances in the SPhP-active (>1380 cm^−1^) spectral region. The spectra from different locations exhibit variations of the PFIR spectra. For data taken close to the terminal of the BNNT (distance, <650 nm), the high-frequency (>1420 cm^−1^) SPhP mode appears in the PFIR spectra. At locations far from the terminal, the high-frequency SPhP mode gradually disappears. This observation can be explained by the fact that high-frequency SPhP decays faster and, therefore, is less detectable when the probe is far from the terminal (*49*). Whereas in s-SNOM the spatial contrast of SPhP is attributed to the reflection of SPhPs from the terminal of the BNNT, the generation and detection of SPhPs in PFIR microscopy are due to the enhanced local infrared absorption of the nanotube in the presence of the metal-coated probe.

One possible mechanism of the generation and detection of SPhPs in PFIR microscopy on the BNNT is the field-enhancement effect of the metal-coated probe. In the peak force tapping cycle, the sharp metal-coated probe is in controlled, small-depth contact with the surface by a small area, less than the radius of the probe. In the SPhP-active frequency regime, a donut-shaped field enhancement is formed surrounding the metallic probe, because the real part of the dielectric function of the boron nitride is negative. The field is concentrated with high spatial frequencies that are capable of exciting the SPhPs in the BNNT. The excited SPhPs quickly dissipate and convert into heat in the BNNT that causes thermal expansion, which leads to detectable mechanical responses of the cantilever. The scheme is shown in Fig. 5H. The signal generation scheme shares similar elements with the launch of SPhPs in the BNNT in the case of s-SNOM (shown in Fig. 5I), where the field enhancement and light concentration at the tip apex launch the SPhPs. This is the reason why the PFIR and s-SNOM images at the same frequency show similarities on the overall distribution of SPhPs. The difference is that PFIR monitors the energy loss of the SPhPs that leads to the local thermal dissipation, whereas s-SNOM monitors the effective polarizability of the s-SNOM probe that is modified by the presence of SPhPs of the material.

## DISCUSSION

PFIR microscopy has several advantages over current infrared nanoscopy techniques. In comparison to infrared s-SNOM, PFIR microscopy uses a simple apparatus that does not rely on optical interferometric detection. The signal from PFIR is directly proportional to the infrared absorption, unlike the case of s-SNOM. Both chemical imaging and nanoscale point spectroscopy can be achieved with one simple laser source. In the case of s-SNOM, single-wavelength imaging and nano-FTIR measurement have to be carried out with separate narrowband and broadband laser sources, inevitably adding to the total cost if both capabilities are required.

As an infrared microscopy technique, PFIR is associated with a high spatial resolution. We use the width between 90 and 10% of the maximal height from a nano edge as the estimation of spatial resolution. Figures 3I, 4F, and 5J show signal profiles across sharp features on the PFIR images, and a consistent spatial resolution of about 10 nm is observed in all cases regardless of type or thickness of the sample. In a detailed scan of the block copolymer of 400 nm × 400 nm, we have observed a spatial resolution of 6 nm (fig. S6). The spatial resolution is, in fact, better than the radius of the probe, which is characterized to be about 30 nm using a standard tip-quantification procedure by the NanoScope Analysis software of the Multimode AFM (fig. S7). Spatial resolution smaller than the tip radius is seen because the size of the contact area in peak force tapping can be smaller than the tip radius if the indentation depth is small. In our measurement, we use a peak force amplitude set point of 4 nN, and the deformation of the sample surface is found to be 0.3 ± 0.1 nm. Assuming a rigid, perfect sphere with a radius of 30 nm in contact, only a circular area with a radius of 3 ± 0.5 nm is in contact with the sample surface, which determines the limit of the spatial resolution, if the laser-induced mechanical responses are locally generated and detected. The improved spatial resolution of PFIR is clearly demonstrated in Fig. 5 by the comparison between the PFIR images and the s-SNOM images, where PFIR images show a much higher spatial resolution, although an identical AFM probe is used. We have also found that reducing the peak force set point within the operational range of the peak force tapping increases the spatial resolution (fig. S8).

Even with the signal readout from the cantilever contact resonance, PFIR microscopy has a much higher spatial resolution than the contact mode–based PTIR techniques, which reaches 50 to 100 nm for typical samples. In the contact mode PTIR technique, the cantilever *Q* factor for the contact resonance has two opposing effects. On the one hand, a high *Q* factor provides strong contact resonance signal; on the other hand, a high *Q* factor means a high amount of residual contact resonance oscillations generated from adjacent locations due to thermal diffusion. The residual oscillations from adjacent locations lead to the convolution of signals and the reduction of spatial resolutions. In PFIR microscopy, the fast and periodical peak tapping cycles allow the use of a high *Q* factor cantilever during the controlled contact to exhibit a strong amplitude of contact resonance and, afterward, effectively dampens the residual contact resonance oscillations when the probe is retracted from the sample surface. This is because the *Q* factor of the contact resonance becomes very low when the tip is detached from the surface, also ensuring that each contact in the peak force tapping cycle is fresh, that is, not affected by contact resonance oscillations of previous cycles. This feature bypasses the dilemma of *Q* factors in the PTIR technique. In the case of signal readout from the baseline offset, the peak force tapping allows the readout of laser-induced volume expansions from a short period of 20 μs or less, which is a time scale comparable to or smaller than the characteristic thermal diffusion time (*51*). Consequently, the measurement right after the pulsed laser excitation in PFIR transduces the response of the material before thermal diffusion. The reduction of spatial resolution due to thermal diffusion is effectively alleviated for both data extraction methods.

PFIR microscopy inherits the wide applicability of the peak force tapping mode. Peak force tapping mode has been proven to be robust on various rough and sticky sample surfaces that are not suitable for the contact mode or regular tapping mode AFM operations (*52*). This attribute is a practical advantage of PFIR over existing contact mode– or tapping mode–based infrared scanning probe microscopy techniques, such as s-SNOM, PiFM, or the tapping mode AFM-IR. Compared to PiFM as a nanoscale infrared imaging technique, the PFIR technique does not require the probe cantilever to have two well-behaved mechanical resonances. In the peak force tapping mode, the free-space mechanical resonance of the cantilever is not intentionally excited or used. Therefore, the PFIR technique is open to a wider range of probes than PiFM, providing flexibility for future multimodal measurements that combine other modalities of AFM such as conductivity mapping or Kelvin probe force microscopy.

Another advantage of PFIR microscopy is its low average power requirements. In our implementation, only a 20-μW power is used for a peak force tapping rate of 2 kHz. Even if the peak force tapping rate is further increased to 8 kHz, the average power of the laser is still below 100 μW. At this power level, the laser-induced thermal damage of the sample is negligible. The temperature increase due to laser exposure is exceedingly small. This property is an advantage for measurement that requires a constant temperature. In comparison, for s-SNOM, milliwatt-level average power is required for imaging and spectroscopy, which may cause sample damages for thermally metastable samples.

PFIR microscopy allows simultaneous mechanical and chemical measurements in a single measurement with one simple apparatus, a feature that has not yet been demonstrated by other nanoscale characterization techniques. The simultaneous multimodal measurement provides better correlations than sequential measurement, because it does not suffer from the sample position drift associated with sequential measurement. The ability to perform multimodal correlative measurements is particularly suitable in the investigations of complex materials without a priori knowledge of their structures.

The speed of PFIR microscopy scales linearly with the operational speed of the peak force tapping. Current acquisition of a PFIR image of 256 × 256 pixels requires about 30 min at a 2-kHz peak force tapping frequency. If the peak force tapping frequency can be increased to 8 kHz, which is possible, the time will be reduced to 7.5 min, which is a typical time frame for a scanning probe microscopy scan. The limiting factor of the method is the frequency coverage of light source. The spectral coverage of our apparatus is limited by the tunability of the QCL to several hundreds of wave numbers. If a light source with a wide tunability range is used, such as solid-state lasers that can generate frequency-tunable infrared pulses through nonlinear parametric processes, then the PFIR microscopy will provide more information in studying the compositions of unknown samples and associated chemical transformations.

In summary, the method of PFIR microscopy enables reliable chemical imaging, the collection of broadband spectra, and simultaneous mechanical mapping in one simple setup with a spatial resolution of ~10 nm. We have investigated three types of representative materials, namely, soft polymers, perovskite crystals, and BNNTs, all of which provide a strong PFIR resonance for unambiguous nanochemical identification. Many other materials should be suited as well for the multimodal characterization that PFIR microscopy has to offer. Possible future investigations could include deciphering the relationships between the composition and mechanical properties of biological materials, such as protein fibrils and bones. The high spatial resolution and multimodal characterization ability of PFIR will undoubtedly provide a useful analytical tool for the nanoscience and nanotechnology community.

## MATERIALS AND METHODS

### PFIR microscopy

The apparatus of PFIR microscopy is shown in Fig. 1. It consists of an AFM with peak force tapping capability (PeakForce Tapping, Multimode AFM with NanoScope V controller, Bruker Nano), a QCL (MIRcat, Daylight Photonics), a customized circuit for synchronization and triggering the laser, and a customized optical setup that delivers the focused infrared beam to the region of the tip and sample. Pt-coated cantilever probes (MikroMasch HQ:NSC14/Pt) were used. In PFIR microscopy, the probe taps the sample under the feedback of the peak force tapping mode. The vertical position of the sample was sinusoidally modulated at a frequency of several kilohertz beneath the probe, which is fixed in the stationary position. Around the upper turning point of the sample oscillation, the sample was in controlled contact with the tip of the probe. The maximal vertical deflection amplitude of the cantilever during the contact, known as the peak force, was used as a set point of the feedback to maintain the average distance between the sample and the tip. The mechanical properties of modulus and adhesion were extracted by analyzing the time-varying trace of the vertical deflection of the cantilever with the NanoScope 9.2 software that controls the AFM. In the experiment, the frequency of peak force tapping was set at 2 kHz, that is, the sample and the probe are in controlled, dynamic contact 2000 times per second. The waveform of the peak force tapping frequency was routed to a function generator (FN 81 Fluke) and a customized circuit to generate a phase-locked, frequency-halved, 1-kHz transistor-transistor logic (TTL) waveform, which is used to trigger the QCL that is operated in the pulsed mode. The phase of the TTL waveform controls the timing of the emitted laser pulse in the peak force tapping cycle. The timing was externally adjusted such that the pulse illuminates the tip and sample when they are in the controlled, dynamic contact. The timing of the peak force amplitude feedback was set to be slightly ahead of the arrival of the laser pulse such that the laser-induced mechanical effects of the cantilever do not affect the feedback of peak force tapping. Because the repetition rate of the laser pulses is triggered at half of the peak force tapping frequency, laser pulses interact with the tip and sample in every other peak force tapping cycle. The pulse duration of the infrared laser was set at 20 ns. At the triggering rate of 1 kHz, the average power of the laser is between 5 and 20 μW, which corresponds to a per pulse energy in the range of 5 to 20 nJ. The infrared beam from the QCL was expanded to a diameter of 12 mm by a reflective beam expander. The laser beam was focused at the sample and tip region with an off-axis parabolic mirror with polarization parallel to the tip axis. Diagnostic optics with auxiliary He-Ne lasers were used to ensure that the infrared beam was focused properly to the tip region. The signal from the vertical deflection of the AFM cantilever was read out by a quadrant photodiode of a 5-MHz readout bandwidth and routed to a data acquisition card (PXI-5122, National Instruments) to be recorded at every peak force tapping cycle. Because the laser interacts with the sample only every other peak force tapping cycle, two types of traces of tip vertical deflection are obtained and processed: (i) first is the tip vertical deflection with synchronized pulsed illumination, and (ii) the second is acquired without the presence of the laser and used as the reference background. The PFIR trace results from subtraction of the two traces. To increase the signal-to-noise ratio, many PFIR traces were averaged in the gate-triggered signal acquisition. In PFIR imaging, 50 peak force tapping cycles were averaged for each pixel. In the point spectroscopy mode, 1000 peak force tapping cycles were acquired for each wave number step. The amplitude of the cantilever contact resonance oscillation was extracted from the oscillations in the PFIR trace by numerical fast Fourier transforms using a LabVIEW program. The baseline offset of the PFIR trace was extracted by the averaged difference between the baselines before and after the laser interaction with the same LabVIEW program. Both the amplitude of contact resonance oscillation and the baseline offset were used as PFIR signals. We find that the two types of PFIR signals provide similar results on nanoscale imaging of soft materials (figs. S4 and S6) as well as point spectra, although for rigid samples, cantilever contact resonance provides better results than the baseline offset.

In the imaging mode of PFIR microscopy, the wavelength of the QCL was fixed to match an infrared resonance of the sample. The PFIR signals were recorded together with topography, modulus, and adhesion while the AFM is scanning over the sample in the peak force tapping mode. The peak force set point in the peak force tapping was chosen to be several nanonewtons, which corresponds to the maximal force of the cantilever applied to the sample. The value of the peak force set point does not affect the shape of the infrared spectrum (fig. S9); but a low set point reduces the amplitude readout from the contact resonance, because the cantilever is not efficiently anchored on the surface with a low peak force set point. A peak force set point of 4 nN was found to be good for the measurement using the MikroMasch HQ:NSC14/Pt probe with a spring constant of 5 N/m. In the point spectroscopy mode of PFIR microscopy, the probe of the AFM stayed at a fixed location while maintaining the peak force tapping feedback. The frequency of the QCL was scanned. At each frequency, the PFIR signals were recorded to form the PFIR spectra that are proportional to the infrared absorption spectra. The frequencies of the QCL were tuned across the spectral range while avoiding strong water absorption lines in the infrared fingerprint region. The power of the laser at different wavelengths was measured with a mercury cadmium telluride (MCT) detector (KLD-0.25-FJ1/AC5M/30/11, Kolmar Technologies) or with reference nonresonant samples and used to normalize the amplitude-collected PFIR spectra to compensate for residual water absorption or nonuniform power over the QCL tuning range.

The mechanical measurement was performed with the peak force tapping simultaneously with the PFIR signal extraction. The real-time indentation of the sample was averaged, analyzed, and fitted with the DMT model (*40*) to extract the modulus of the sample as well as the adhesion between the probe and the sample. The extractions of the quantitative mechanical properties were done with the standard operation software of the Multimode AFM (NanoScope 9.2, Bruker Nano). Parameters including the radius of the probe, the spring constant of the cantilever, and the deflection sensitivity of the cantilever were characterized with standard procedures. We find that the presence of the laser pulse does not affect the values of modulus and adhesion of the sample, so long as the laser energy is kept in the linear regime.

### Scattering-type scanning near-field optical microscopy

The s-SNOM measurement in Fig. 5 (E and F) was done with a customized scattering near-field optical microscope that shares the same AFM as the PFIR apparatus. The setup of the s-SNOM was built according to the study by Xu *et al*. (*53*). In the s-SNOM measurement, the metal-coated probe (MikroMasch HQ:NSC14/Pt) was modulated in tapping mode over the sample of interest. The infrared laser from the QCL (MIRcat, Daylight Solutions) was operated in continuous wave with an output of about 40 to 70 mW and guided onto the tip and sample region using a parabolic mirror with a 25-mm effective focal distance. The scattered light was collected by the same parabolic mirror and optically homodyned with a Michelson interferometer. The homodyned scattering infrared field was detected by the same MCT detector. The electric signal from the detector was coupled to a lock-in amplifier (HF2LI, Zurich Instruments) to obtain the third harmonic demodulation with the tapping frequency of the probe as the reference frequency. The demodulation signal was used as the s-SNOM signal. The in-phase homodyne condition was used by positioning the reference arm of the Michelson interferometer to maximize the s-SNOM signal from a nonresonant substrate in the BNNT measurement.

### Sample preparation

The P2VP sample (P19124A-2VP, Polymer Source) was prepared by drop-casting the solution of P2VP in toluene on a gold substrate. The PS-*b*-PMMA sample (95-*b*-92; P8537-SMMA, Polymer Source) was dissolved in toluene and spin-coated on a gold substrate. The spin-coater (KW-4A) uses 620 revolutions per minute (rpm) for 9 s and 2560 rpm for 20 s. The thickness of the block copolymer was found to be 30 nm. The perovskite sample was synthesized according to the procedures described in the study by Yang *et al*. (*54*). The perovskite crystal was about 3 mm × 3 mm in size and 2 mm in thickness. The surface of the orange-colored single crystal of CH_3_NH_3_PbBr_3_ was measured with the PFIR microscopy. The BNNTs were used as received. After being dispersed with isopropanol and sonicated, BNNTs were drop-casted on a gold substrate. The BNNTs were synthesized via chemical vapor deposition (*55*).

## Supplementary Material

http://advances.sciencemag.org/cgi/content/full/3/6/e1700255/DC1

## SUPPLEMENTARY MATERIALS

Supplementary material for this article is available at http://advances.sciencemag.org/cgi/content/full/3/6/e1700255/DC1 fig. S1. Fourier transform of the PFIR trace. fig. S2. Power dependence of the baseline offset amplitude. fig. S3. Mechanical behaviors and power dependence of PMMA. fig. S4. Comparison between the cantilever oscillation amplitude and baseline offset in PFIR microscopy on the PS-*b*-PMMA block copolymer. fig. S5. Deformation map of perovskite from peak force tapping microscopy. fig. S6. Detailed PFIR image of a 400-nm × 400-nm region of the PS-*b*-PMMA block copolymer. fig. S7. Procedure to calibrate the tip radius for PFIR microscopy. fig. S8. PFIR images with different peak force set points. fig. S9. The spectra of PTFE with different peak force set points.
