# Supplementary material for: Nanoscale simultaneous chemical and mechanical imaging via peak force infrared microscopy
Source: Sci Adv. 2017 Jun 23;3(6):e1700255. doi: 10.1126/sciadv.1700255 (PMC5482550; doi:10.1126/sciadv.1700255)
Supplement: http://advances.sciencemag.org/cgi/content/full/3/6/e1700255/DC1 [file supp_3_6_e1700255__index.html]

Science Advances | Science Advances

## Supplementary Materials

**This PDF file includes:**

- fig. S1. Fourier transform of the PFIR trace.
- fig. S2. Power dependence of the baseline offset amplitude.
- fig. S3. Mechanical behaviors and power dependence of PMMA.
- fig. S4. Comparison between the cantilever oscillation amplitude and baseline offset in PFIR microscopy on the PS-*b*-PMMA block copolymer.
- fig. S5. Deformation map of perovskite from peak force tapping microscopy.
- fig. S6. Detailed PFIR image of a 400-nm × 400-nm region of the PS-*b*-PMMA block copolymer.
- fig. S7. Procedure to calibrate the tip radius for PFIR microscopy.
- fig. S8. PFIR images with different peak force set points.
- fig. S9. The spectra of PTFE with different peak force set points.

Download PDF

**Files in this Data Supplement:**

- Adobe PDF - 1700255\_SM.pdf
