# Supplementary material for: Nanoscale simultaneous chemical and mechanical imaging via peak force infrared microscopy
Source: Sci Adv. 2017 Jun 23;3(6):e1700255. doi: 10.1126/sciadv.1700255 (PMC5482550; doi:10.1126/sciadv.1700255)
Supplement: http://advances.sciencemag.org/cgi/content/full/3/6/e1700255/DC1 [file 1700255_SM.pdf]

Science Advances

AAAS

advances.sciencemag.org/cgi/content/full/3/6/e1700255/DC1

Supplementary Materials for

Nanoscale simultaneous chemical and mechanical imaging via peak force infrared microscopy

Le Wang, Haomin Wang, Martin Wagner, Yong Yan, Devon S. Jakob, Xiaoji G. Xu

Published 23 June 2017, *Sci. Adv.* **3**, e1700255 (2017)
DOI: 10.1126/sciadv.1700255

This PDF file includes:

- fig. S1. Fourier transform of the PFIR trace.
- fig. S2. Power dependence of the baseline offset amplitude.
- fig. S3. Mechanical behaviors and power dependence of PMMA.
- fig. S4. Comparison between the cantilever oscillation amplitude and baseline offset in PFIR microscopy on the PS-*b*-PMMA block copolymer.
- fig. S5. Deformation map of perovskite from peak force tapping microscopy.
- fig. S6. Detailed PFIR image of a 400-nm × 400-nm region of the PS-*b*-PMMA block copolymer.
- fig. S7. Procedure to calibrate the tip radius for PFIR microscopy.
- fig. S8. PFIR images with different peak force set points.
- fig. S9. The spectra of PTFE with different peak force set points.

Supplementary Materials

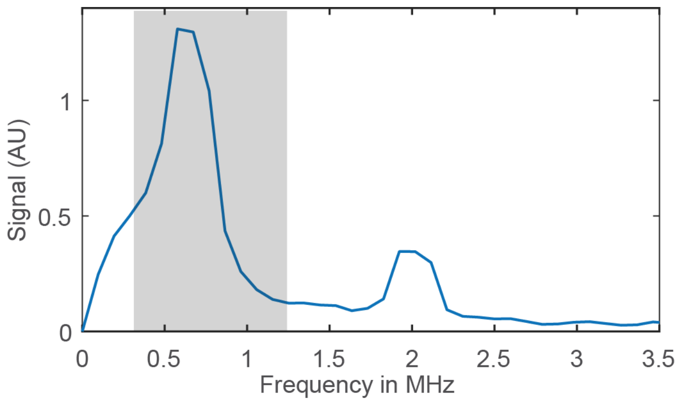

fig. S1. Fourier transform of the PFIR trace. Fourier transform of the oscillation part of the PFIR trace reveals the contact resonance oscillation of the cantilever (Mikromash HQ:NSC14/Pt), which is excited by the absorption of the infrared pulse and subsequent volume expansions. The frequency of the contact resonance at 620 kHz is found to be much higher than the free space mechanical resonant frequency of 150 kHz. In the oscillation read out, the total area under the first contract resonance peak centered at 620 kHz (marked by the gray region) is obtained and used as the PFIR signal. The width of the area summation is set relatively large, because the contact resonance may change for different materials.

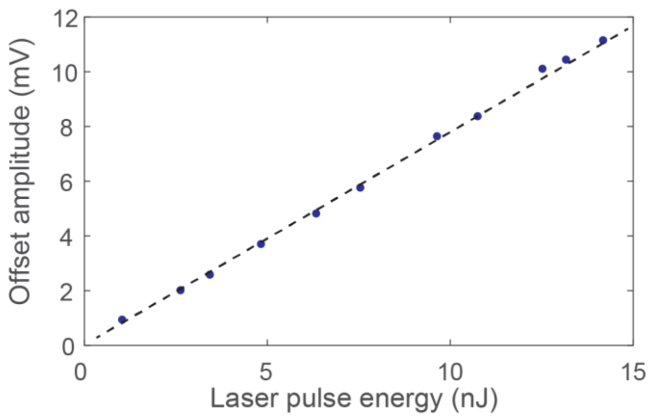

fig. S2. Power dependence of the baseline offset amplitude. The sample is PTFE, and the infrared frequency is 1210 cm^{-1}, on resonance with the PTFE absorption. The power dependence shows an overall linear behavior, similar to the power dependence shown in Figure 1e of the main article.

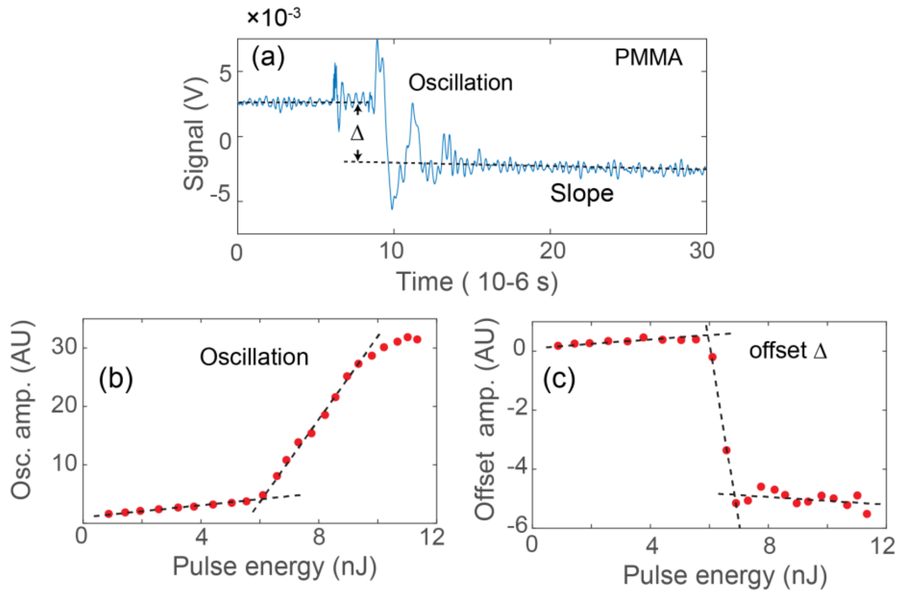

fig. S3. Mechanical behaviors and power dependence of PMMA. (**a**) Laser induced cantilever behaviours on the sample of PMMA polymer in the peak force tapping mode. The laser is tuned to the frequency of 1725 cm^{-1}, on resonance with the PMMA absorption. The mechanical oscillation and baseline offset are observed. The mechanical oscillation is attributed to the excitation of the contact resonance of the cantilever. The baseline offset is from the change of the height of the sample surface. The downward offset suggests contraction of the sample volume after the infrared laser pulse. (**b**) shows the relation between laser power versus the oscillation amplitude of the PFIR trace of the PMMA sample at the resonant infrared frequency of 1725 cm^{-1}. Two linear relations are observed in the low-energy regime and high energy regime. The discontinuity of the power dependence suggests the presence of a phase transition, likely the glass transition of the polymer induced by the absorption of laser energy.

In addition to the baseline offset, a slope is observed for PMMA. The presence of the slope suggests the change of the modulus after the laser excitation. It suggests the conversion between the solid state of the PMMA polymer to a glass state with reduced modulus.

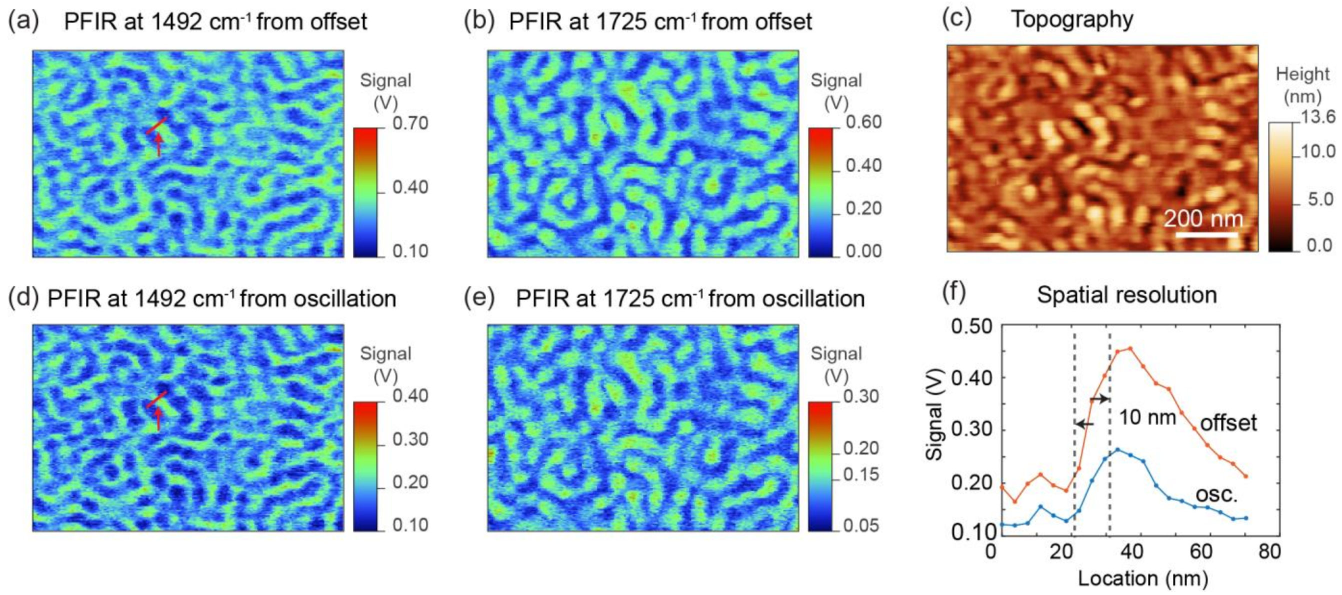

fig. S4. Comparison between the cantilever oscillation amplitude and baseline offset in PFIR microscopy on the PS-*b*-PMMA block copolymer. (**a** to **b**) show the peak force infrared (PFIR) images of the PS-b-PMMA block copolymer at 1492 cm^{-1} and 1725 cm^{-1} respectively. 1492 cm^{-1} matches the infrared absorption of polystyrene; 1725 cm^{-1} matches the infrared absorption of PMMA. Both PFIR images are formed by registration of the amplitude of the laser induced baseline offset on the polymer sample. The topography of the area is shown in (**c**). The co-registered PFIR image of the same area and same infrared frequencies but based on the integrated amplitude of the contact resonance is shown in (**d** to **e**). The images are the same as in Figure 3 (b-c) of the main article. It can be seen that both the integrated amplitude of the contact resonance and the amplitude of the laser-induced baseline shift can be used for chemical sensitive imaging. (**f**) Cut profiles from the PFIR images of (a) and (d) from the same locations (marked by the red line). The profile from the PFIR signal through laser-induced baseline offset (red curve) show a similar spatial contrast to that of PFIR signal through the amplitude of the contact resonance oscillation (blue curve). In both cases, spatial resolutions of 10 nm are observed, estimated from the width between 10% to 90% of the edge height.

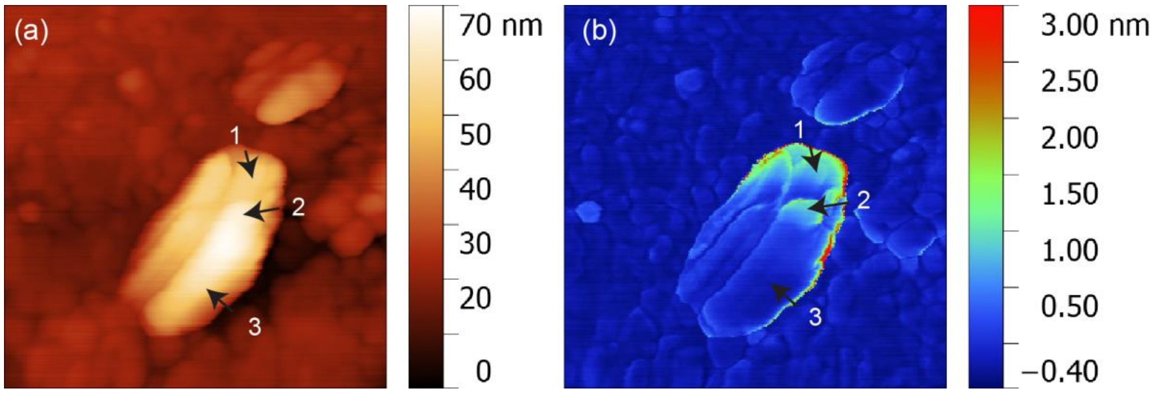

fig. S5. Deformation map of perovskite from peak force tapping microscopy. (**a**) The topography of the perovskite nanocrystal studied in the main article (the same image as Figure 4a). (**b**) The deformation map of the

same area, co-registered during PFIR microscopy. Location 2 shows clearly different deformation depth from the lower part of the nanocrystal, indicating the sub-domain at location 2 is different from the main nanocrystal.

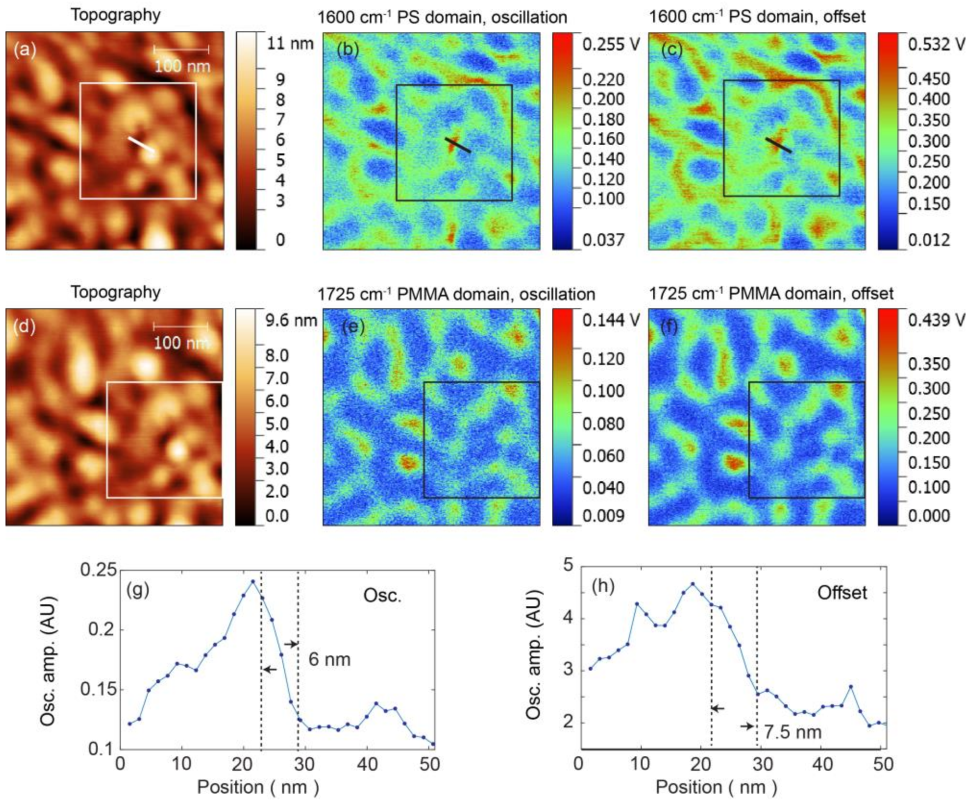

fig. S6. Detailed PFIR image of a 400-nm × 400-nm region of the PS-*b*-PMMA block copolymer. (**a**) Topography of the surface of the PS-b-PMMA sample used in the main article. (**b**) PFIR image based on the amplitude of the contact resonance oscillation. The laser frequency is tuned to the resonant frequency of polystyrene domain at 1600 cm^{-1}. (**c**) PFIR image based on the value of laser-induced baseline shift. The laser frequency is 1600 cm^{-1}. (**d**) Topography of the same block copolymer surface with a small position drift. Square boxes on the images are used to indicate the same area on the block-copolymer (**e**) PFIR image based on the amplitude of the contact resonance oscillation. The laser frequency is tuned to the resonant frequency of PMMA domain at 1725 cm^{-1}. (**f**) PFIR image based on the value of laser-induced baseline shift. The laser frequency is 1725 cm^{-1}. Note that the infrared power was reduced for PMMA to 15% of that of polystyrene to avoid the phase transition of PMMA. (**g** to **h**) Cut profiles from PFIR images of oscillation amplitude and laser-induced baseline offset. A spatial resolution of 6 nm from PFIR with contact resonance oscillation amplitude and 7 nm from the PFIR image of laser induced-baseline offset are observed, respectively. The spatial resolutions are estimated from the width between 90% and 10% of the edge height.

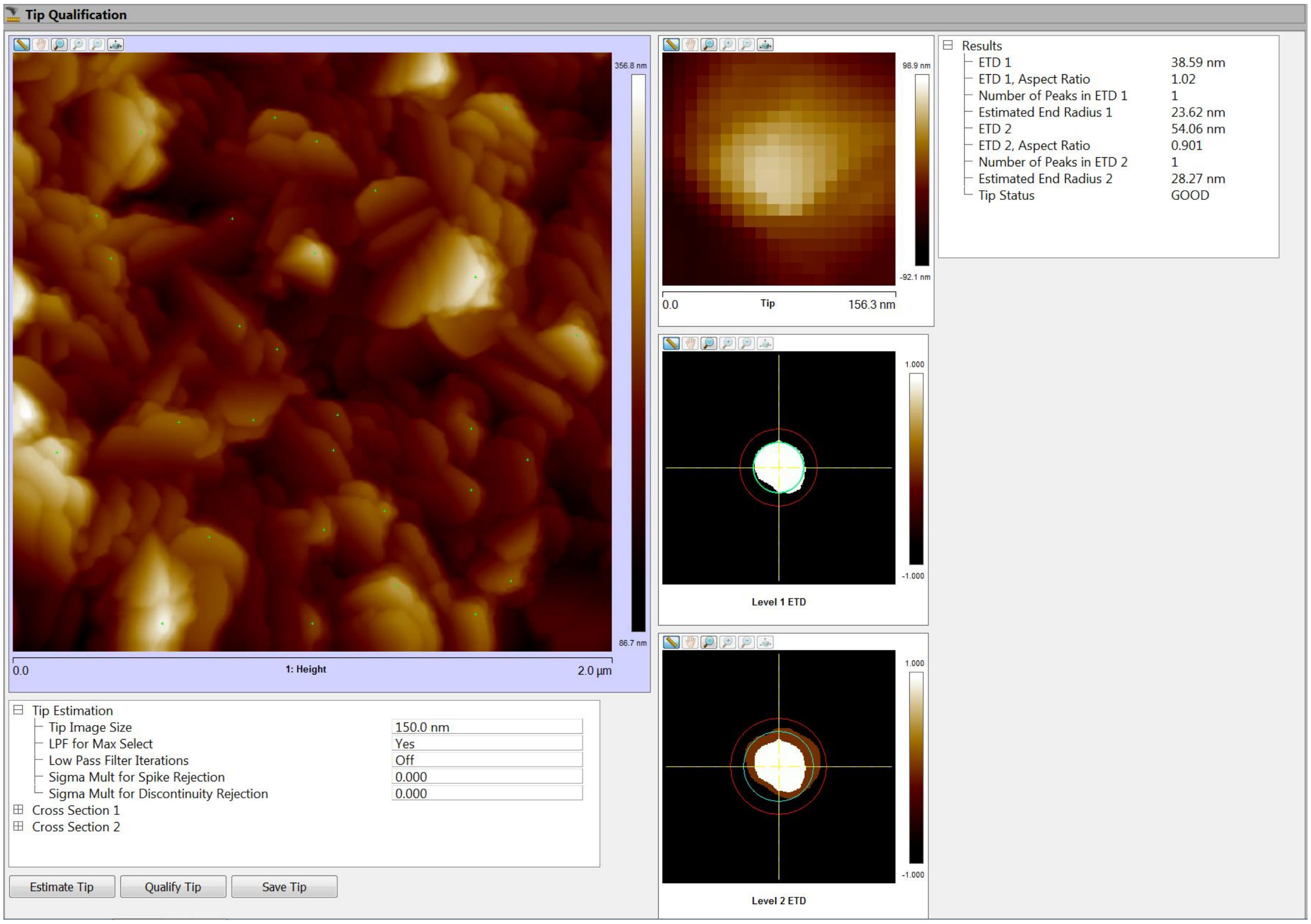

fig. S7. Procedure to calibrate the tip radius for PFIR microscopy. The peak force tapping topography is collected from a rough TiO_2 sample with sharp features. The Bruker Nanoscope Analysis software (version 1.8) has a function to recover the tip radius. It involves filtering of spatial frequencies and the deconvolution of the shape of the tip. The radius is used as a parameter of the peak force tapping. This procedure is recommended as part of the routine operation for peak force tapping. Here an unused tip is quantified with the procedure, and an end radius of 28.3 nm is found.

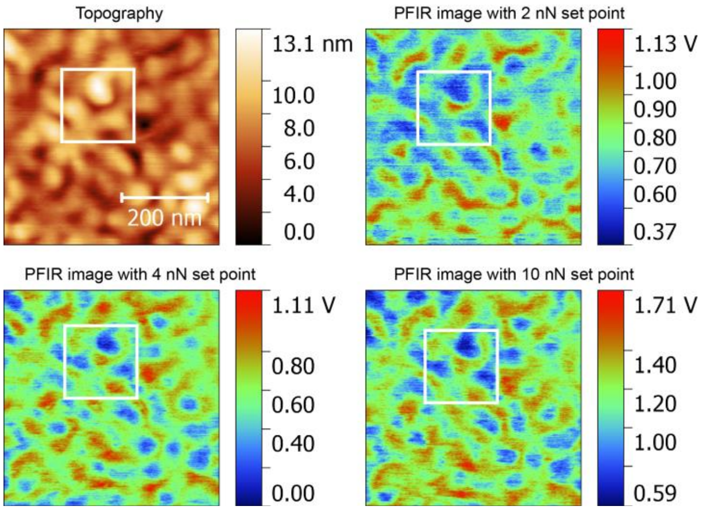

fig. S8. PFIR images with different peak force set points. We have carried out PFIR experiment on the PS-b-PMMA block-copolymers with three peak force set points of 2 nN, 4 nN, and 10 nN. We find that a lower peak force set point improves the spatial resolution, as the PFIR images are sharper. The white boxes mark the same region across the images. The observation can be interpreted as the reduction of the contact area with low peak force set point. The smaller contact area in the peak force tapping reduces the area the mechanical signal is detected, therefore increases the spatial resolution.

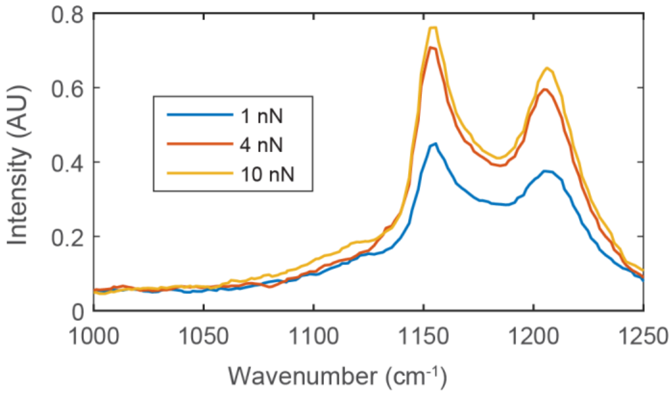

fig. S9. The spectra of PTFE with different peak force set points. The set point of 1 nN, 4 nN and 10 nN are used. The spectra are based on the amplitude of the contact resonance v.s. the infrared frequency. All set points give similar shape of the infrared spectral profiles. It can be seen that the amplitude of the contact resonance oscillation is reduced if the peak force set point is set too small, i.e., 1 nN. The peak force set point of 4 nN and 10 nN give roughly the same amplitude.

Supplementary Materials

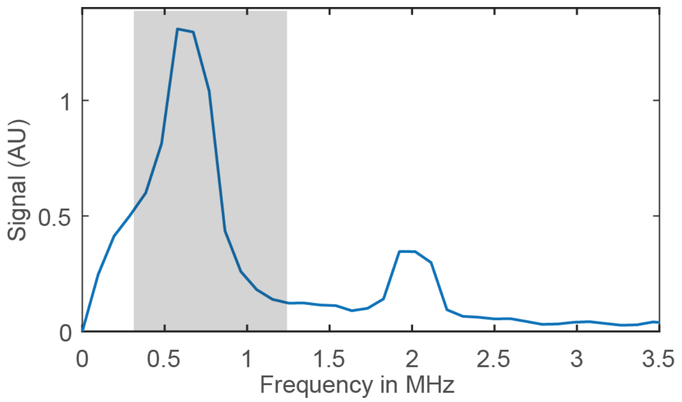

fig. S1. Fourier transform of the PFIR trace. Fourier transform of the oscillation part of the PFIR trace reveals the contact resonance oscillation of the cantilever (MikroMasch HQ:NSC14/Pt), which is excited by the absorption of the infrared pulse and subsequent volume expansions. The frequency of the contact resonance at 620 kHz is found to be much higher than the free space mechanical resonant frequency of 150 kHz. In the oscillation read out, the total area under the first contract resonance peak centered at 620 kHz (marked by the gray region) is obtained and used as the PFIR signal. The width of the area summation is set relatively large, because the contact resonance may change for different materials.

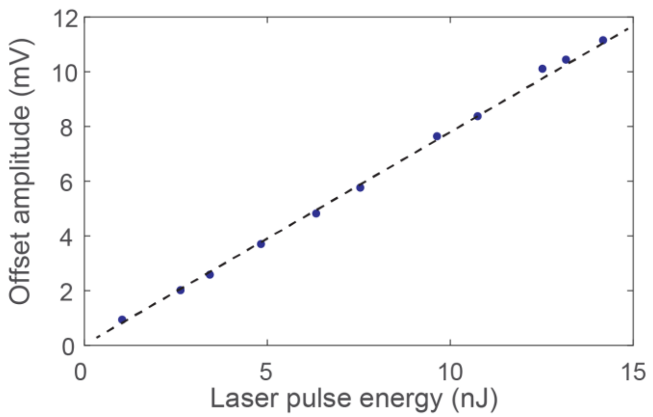

fig. S2. Power dependence of the baseline offset amplitude. The sample is PTFE, and the infrared frequency is 1210 cm^{-1}, on resonance with the PTFE absorption. The power dependence shows an overall linear behavior, similar to the power dependence shown in Fig. 1E of the main article.

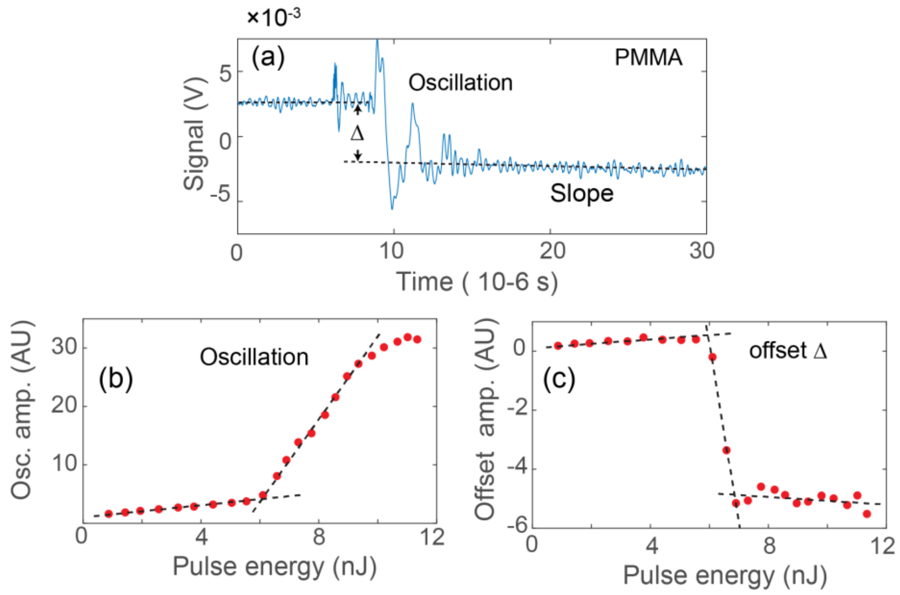

fig. S3. Mechanical behaviors and power dependence of PMMA. (**a**) Laser induced cantilever behaviors on the sample of PMMA polymer in the peak force tapping mode. The laser is tuned to the frequency of 1725 cm^{-1}, on resonance with the PMMA absorption. The mechanical oscillation and baseline offset are observed. The mechanical oscillation is attributed to the excitation of the contact resonance of the cantilever. The baseline offset is from the change of the height of the sample surface. The downward offset suggests contraction of the sample volume after the infrared laser pulse. (**b**) shows the relation between laser power versus the oscillation amplitude of the PFIR trace of the PMMA sample at the resonant infrared frequency of 1725 cm^{-1}. Two linear relations are observed in the low-energy regime and high-energy regime. The discontinuity of the power dependence suggests the presence of a phase transition, likely the glass transition of the polymer induced by the absorption of laser energy.

In addition to the baseline offset, a slope is observed for PMMA. The presence of the slope suggests the change of the modulus after the laser excitation. It suggests the conversion between the solid state of the PMMA polymer to a glass state with reduced modulus.

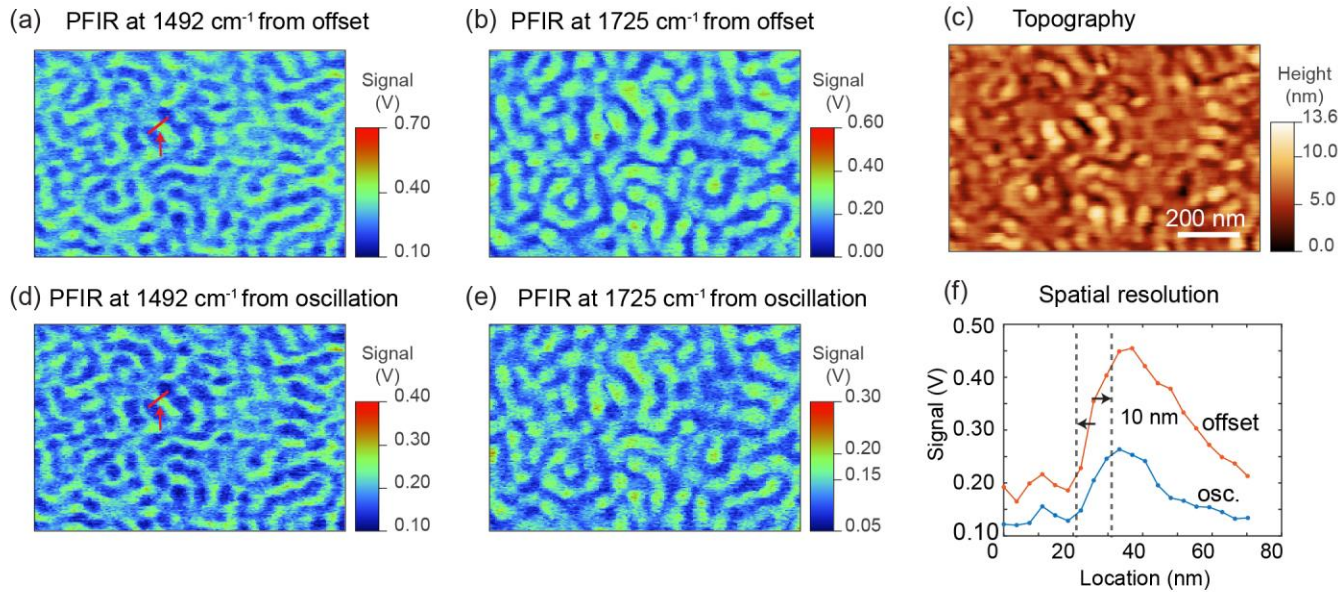

fig. S4. Comparison between the cantilever oscillation amplitude and baseline offset in PFIR microscopy on the PS-*b*-PMMA block copolymer. (**a** to **b**) show the peak force infrared (PFIR) images of the PS-b-PMMA block copolymer at 1492 cm^{-1} and 1725 cm^{-1} respectively. 1492 cm^{-1} matches the infrared absorption of polystyrene; 1725 cm^{-1} matches the infrared absorption of PMMA. Both PFIR images are formed by registration of the amplitude of the laser induced baseline offset on the polymer sample. The topography of the area is shown in (**c**). The co-registered PFIR image of the same area and same infrared frequencies but based on the integrated amplitude of the contact resonance is shown in (**d** to **e**). The images are the same as in Figure 3 (b-c) of the main article. It can be seen that both the integrated amplitude of the contact resonance and the amplitude of the laser-induced baseline shift can be used for chemical sensitive imaging. (**f**) Cut profiles from the PFIR images of (a) and (d) from the same locations (marked by the red line). The profile from the PFIR signal through laser-induced baseline offset (red curve) show a similar spatial contrast to that of PFIR signal through the amplitude of the contact resonance oscillation (blue curve). In both cases, spatial resolutions of 10 nm are observed, estimated from the width between 10% to 90% of the edge height.

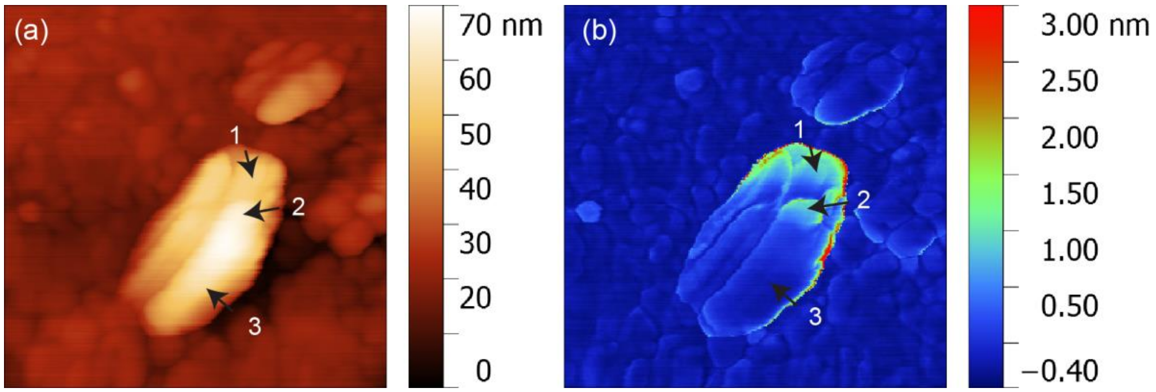

fig. S5. Deformation map of perovskite from peak force tapping microscopy. (**a**) The topography of the perovskite nanocrystal studied in the main article (the same image as Figure 4a). (**b**) The deformation map of the

same area, co-registered during PFIR microscopy. Location 2 shows clearly different deformation depth from the lower part of the nanocrystal, indicating the sub-domain at location 2 is different from the main nanocrystal.

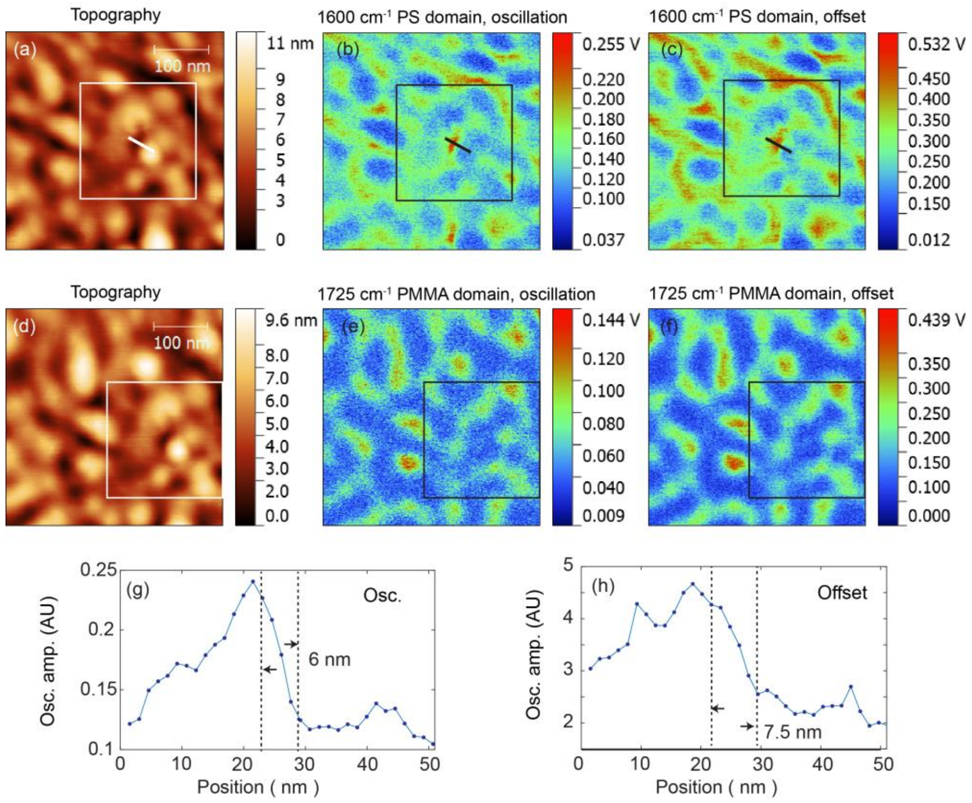

fig. S6. Detailed PFIR image of a 400-nm × 400-nm region of the PS-*b*-PMMA block copolymer. (**a**) Topography of the surface of the PS-b-PMMA sample used in the main article. (**b**) PFIR image based on the amplitude of the contact resonance oscillation. The laser frequency is tuned to the resonant frequency of polystyrene domain at 1600 cm^{-1}. (**c**) PFIR image based on the value of laser-induced baseline shift. The laser frequency is 1600 cm^{-1}. (**d**) Topography of the same block copolymer surface with a small position drift. Square boxes on the images are used to indicate the same area on the block-copolymer (**e**) PFIR image based on the amplitude of the contact resonance oscillation. The laser frequency is tuned to the resonant frequency of PMMA domain at 1725 cm^{-1}. (**f**) PFIR image based on the value of laser-induced baseline shift. The laser frequency is 1725 cm^{-1}. Note that the infrared power was reduced for PMMA to 15% of that of polystyrene to avoid the phase transition of PMMA. (**g** to **h**) Cut profiles from PFIR images of oscillation amplitude and laser-induced baseline offset. A spatial resolution of 6 nm from PFIR with contact resonance oscillation amplitude and 7 nm from the PFIR image of laser induced-baseline offset are observed, respectively. The spatial resolutions are estimated from the width between 90% and 10% of the edge height.

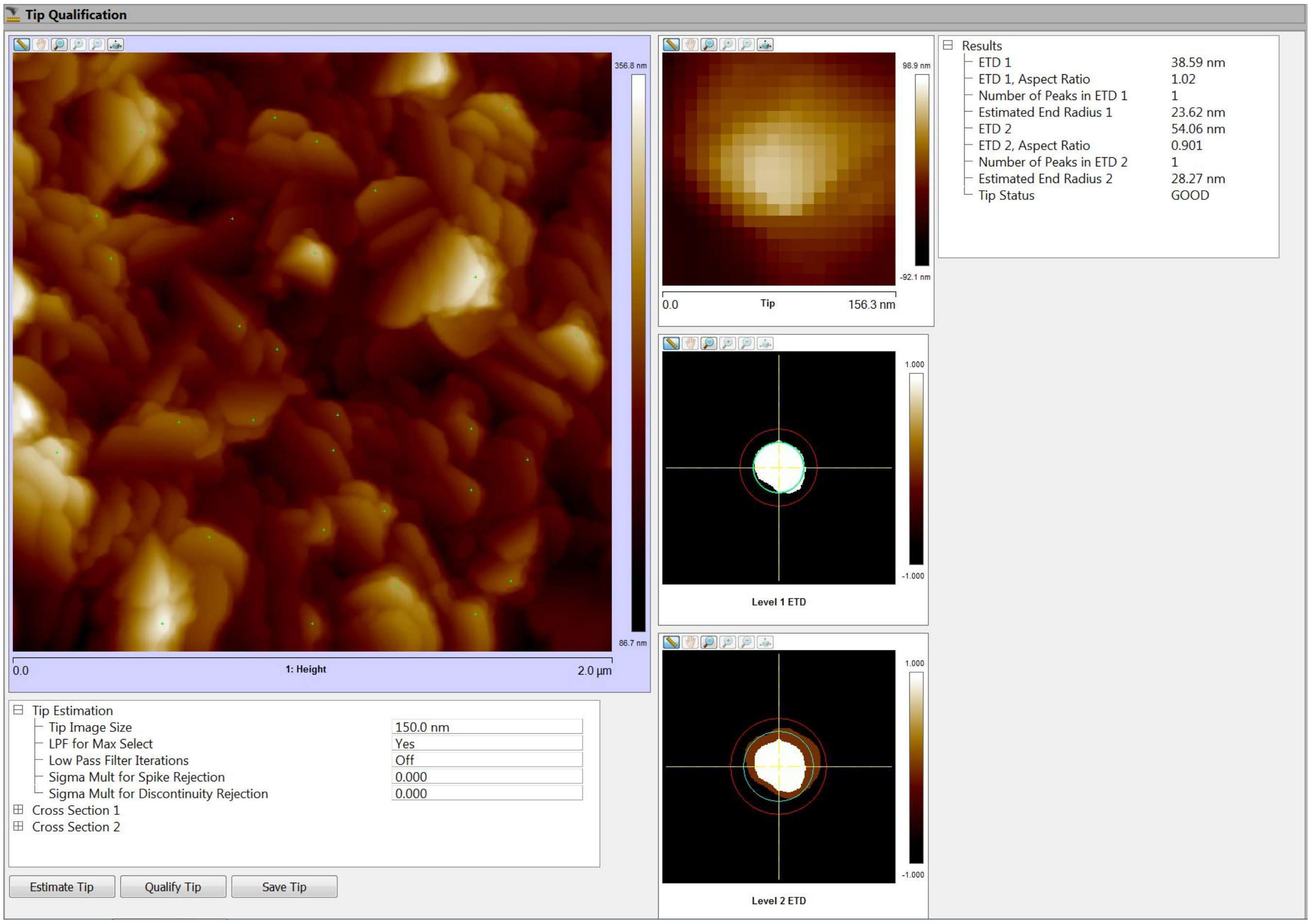

fig. S7. Procedure to calibrate the tip radius for PFIR microscopy. The peak force tapping topography is collected from a rough TiO_2 sample with sharp features. The Bruker NanoScope Analysis software (version 1.8) has a function to recover the tip radius. It involves filtering of spatial frequencies and the deconvolution of the shape of the tip. The radius is used as a parameter of the peak force tapping. This procedure is recommended as part of the routine operation for peak force tapping. Here an unused tip is quantified with the procedure, and an end radius of 28.3 nm is found.

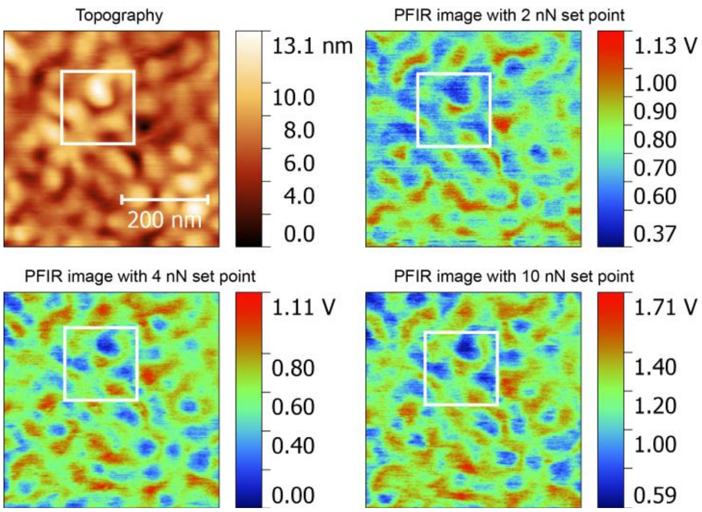

fig. S8. PFIR images with different peak force set points. We have carried out PFIR experiment on the PS-b-PMMA block-copolymers with three peak force set points of 2 nN, 4 nN, and 10 nN. We find that a lower peak force set point improves the spatial resolution, as the PFIR images are sharper. The white boxes mark the same region across the images. The observation can be interpreted as the reduction of the contact area with low peak force set point. The smaller contact area in the peak force tapping reduces the area the mechanical signal is detected, therefore increases the spatial resolution.

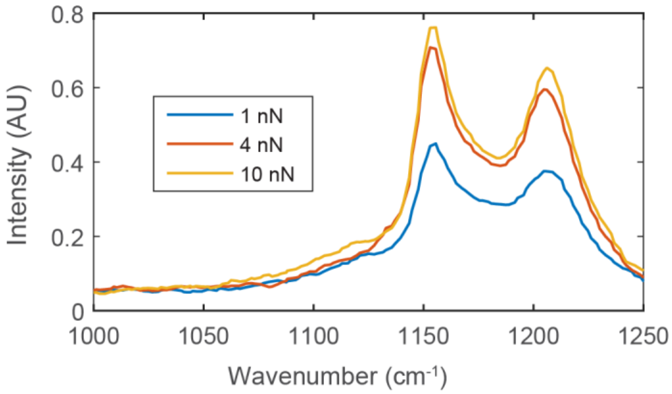

fig. S9. The spectra of PTFE with different peak force set points. The set point of 1 nN, 4 nN and 10 nN are used. The spectra are based on the amplitude of the contact resonance v.s. the infrared frequency. All set points give a similar shape of the infrared spectral profiles. It can be seen that the amplitude of the contact resonance oscillation is reduced if the peak force set point is set too small, i.e., 1 nN. The peak force set point of 4 nN and 10 nN give roughly the same amplitude.
